# Stress perception, coping behaviors and work-privacy conflict of student midwives in times of COVID-19 pandemic: the “Healthy MidStudents” study in Germany

**DOI:** 10.1186/s12913-024-10823-5

**Published:** 2024-05-07

**Authors:** Ilona Efimov, Caroline Johanna Agricola, Albert Nienhaus, Volker Harth, Birgit-Christiane Zyriax, Stefanie Mache

**Affiliations:** 1 Institute for Occupational and Maritime Medicine, University Medical Center Hamburg-Eppendorf, Seewartenstr. 10, 20459 Hamburg, Germany; 2 Midwifery Science Health Care Research and Prevention, Institute for Health Services Research in Dermatology and Nursing, University Medical Center Hamburg-Eppendorf, Martinistr. 52, 20246 Hamburg, Germany; 3 Competence Center for Epidemiology and Health Services Research for Healthcare Professionals, Institute for Health Services Research in Dermatology and Nursing, University Medical Center Hamburg-Eppendorf, Martinistr. 52, 20246 Hamburg, Germany

**Keywords:** Midwifery, Health occupation students, Coping behavior, Psychological stress, Work-life conflict, Parenting, COVID-19 pandemic, Germany

## Abstract

**Background:**

Student midwives deliver care for women under challenging job demands, which may affect their mental health– thus creating a high need for health promotion. Given the lack of research addressing this topic, the aim of this study is to examine the links between stress perception, coping behaviors, work-privacy conflict, and perception of COVID-19 pandemic impact on studies of student midwives in northern Germany.

**Methods:**

Data were collected using a cross-sectional online-survey at nine midwifery study sites in northern Germany from October 2022 to January 2023. 342 student midwives (response rate: 61.3%) were surveyed on stress perception, coping behaviors, work-privacy conflict, and perceived impact of the COVID-19 pandemic on their studies. Descriptive, linear regression and moderation analyses were run to test explorative assumptions.

**Results:**

Results revealed that higher levels of perceived stress were reported by 13.4% of student midwives. Social support (*M* = 13.76, *SD* = 2.19) and active stress coping (*M* = 10.72, *SD* = 2.01) were identified as most prevalent coping behaviors in the present sample. It was found that work-privacy conflict was positively associated with stress perception (*ß* = 0.53, *p* =.001) and maladaptive coping behaviors (alcohol and cigarette consumption: *ß* = 0.14, *p* =.015), and negatively associated with adaptive coping behaviors (positive thinking: *ß* = − 0.25, *p* =.001, social support: *ß* = − 0.23, *p* =.001). Students with children reported significantly lower levels of social support than students without children. 55.6% of student midwives perceived a negative impact of the COVID-19 pandemic on their studies (mostly on lectures, seminars, and contact with fellow students).

**Conclusions:**

Key findings highlighted moderate stress levels among student midwives during theoretical study stage. Based on current research, prevalence of high stress levels among student midwives remains unclear. Given the overall heterogeneous, limited research on student midwives’ stress perception, coping behaviors, work-privacy conflict and perceptions of COVID-19 pandemic impact on studies, implications for research are suggested, e.g. longitudinal studies at different time points and settings and interventional studies. Findings provide a starting point for implementation of workplace health promotion in theoretical and practical stages of midwifery science study programs, e.g. training courses on stress prevention and adaptive coping, and for improvement of working conditions.

**Supplementary Information:**

The online version contains supplementary material available at 10.1186/s12913-024-10823-5.

## Background

Midwives provide care for women throughout pregnancy, childbirth and postpartum as well as provide health promotion, sexual and reproductive health services. Despite the relevance of midwives for healthcare systems and society, a shortage of midwives is evident worldwide [1]. In Germany the number of hospitals with staffing problems has increased from 20% in 2014 to 56% in 2022 [2]. There are also shortages in outpatient care for women during pregnancy or postpartum [3]. In addition, 72% of midwives employed in German hospitals work part-time [4]. Given the combination of staff shortage and high job demands, midwives’ as well as student midwives’ working conditions are becoming even more challenging and thus linked to, e.g. impaired health, aspirations to leave the profession and a higher intention to change employment [5–7]. In order to optimize working conditions and consequently maintain health and ensure adequate care for pregnant women, research is needed on the situation of future midwives in Germany. However, according to the current state of research, there are no national studies and only a few international studies on the working and health situation of student midwives [6, 8, 9].

### Theoretical background

We based this study on the transactional stress model according to Lazarus and Folkman [10], an internationally widely-used, well-established theoretical model in research across multiple fields [11]. It was considered suitable for our study given that previous studies with student midwives were also based on this theoretical model [12, 13]. This process-oriented psychological model of stress and coping describes how different situations and stimuli are perceived and processed by individuals. Psychological stress is defined as “a relationship between the person and the environment that is appraised by the person as taxing or exceeding his or her resources and endangering his or her well-being. The judgement that a particular person-environment relationship is stressful hinges on cognitive appraisal.” [10, p. 21]. The cognitive appraisal process described in the transactional stress model has two phases. In the first step, the relevance of the stimulus is evaluated on the basis of a primary evaluation (irrelevant, positive, or stressful), followed by an appraisal of the consequences, i.e., whether harm or loss has already occurred, whether threat is imminent, or whether the challenge can potentially be met. Threat and challenge represent separate constructs that can also occur simultaneously. In the second step, the available coping resources are assessed, e.g. skills acquired from previous stressful situations, social support or material resources. Both evaluation steps may overlap or influence each other and are not always conscious. Reappraisal may follow if new information has emerged from the environment and/or the individual. Subsequently, individuals may apply two types of coping actions, which either aim at managing or altering the problem causing distress (problem-focused coping) or at regulating the emotional response to the problem (emotion-focused coping). Both can facilitate and hinder each other. Overall, the coping process is characterized by dynamics and change as it is influenced by continuous appraisals and reappraisals of the shifting person-environment relationship (e.g. results of coping efforts) [10].

### Stress perception among student midwives

Current international research indicates that student midwives suffer from psychological strain [6, 8, 9]. Although the prevalence of negative states of mental health among student midwives is inconsistent, several studies exist on the causes and consequences of high psychological strain [8].

On the one hand, student midwives are predominantly exposed to stressors in the clinical setting. In terms of work content, high emotional job demands, e.g., dealing with expectant mothers [14], professional and emotional care in critical situations [15], or with traumatic experiences [6, 16] are mentioned. Students report fears of doing harm during their professional practice and associated negative consequences, as well as fears of attending childbirth professionally for the first time [17]. Students often feel closer to childbearing women than to their colleagues, and thereby experience role conflicts as well as fears of expressing themselves in prescribed procedures [16]. In terms of work organization, on-call [18], time pressure, high workload [19, 20], consequently working overtime [21] and the clinical work environment with inflexible processes and limited opportunities to engage in a counseling role for women are reported as challenges [9, 16]. In addition, social relationships within the clinical setting may also pose stressors for students, e.g., a dysfunctional, unsupportive work culture [16], expectations and relationships with clinical staff [20, 22], little acceptance and appreciation by supervisors and colleagues [16], as well as difficult communication and humiliating experiences by clinical supervisors or instructors (e.g., admonishment in presence of clinical staff) [15] or competition among fellow students [20]. Students report not having a safe space for sharing distressing, traumatic experiences as well as fears and concerns [16].

On the other hand, stressors in the academic setting are also described, including high demands in the course of studies, such as extensive knowledge acquisition for safe professional practice while having insufficient time resources [18, 19], atypical teaching and job schedules [18], little vacation leave [14], interaction with faculty, and aspects of learning environments in higher education institutions [19].

Last, student midwives also experience personal stressors, inter alia, financial concerns [14, 18, 20] or conflicts with extracurricular activities (e.g. engagement in student associations) [14, 20]. In addition, studies also identified family responsibilities [14, 18] and/or maintaining a work-life balance and balancing work/study-related and personal demands as stressors of student midwives [19, 23, 24]. Although no studies exist on parenthood among German student midwives, studies suggest a higher proportion of student midwives with children (15.7%) [25] than in other study programs (total proportion of students with children in Germany is 6%) [26].

### Coping behaviors among student midwives

Emotional exhaustion, depression, and burnout can be psychological consequences of high levels of stress at work [17, 27, 28]. In addition, physiological consequences of stress, such as heart palpitations, fatigue and dizziness, can also be experienced by student midwives [22]. A high experience of stress may also be related to an intention to quit [21]. Few studies indicated that student midwives, on the one hand, exhibit detrimental, maladaptive behaviors in response to high psychological stress in the workplace, such as physical inactivity, unhealthy diets, and alcohol, tobacco, and cannabis use [28], and decreased attention to recreation [18]. On the other hand, further studies with student midwives indicated that also adaptive coping behaviors are applied in the face of high psychological stress [9], such as active coping, the use of emotional support, and positive appraisal [30, 31]. Thereby, the use of adaptive or maladaptive coping behaviors depends on student’s circumstances, needs, and interrelated coping opportunities [9]. In light of the fact that student midwives represent a psychologically taxing as well as socially relevant profession, and are expected to maintain a significant role as health promoters in the future [29], research on their stress perception and coping behaviors is of high relevance. Given a higher proportion of student midwives with children (compared to other study programs) [25, 26], we examine following explorative assumptions:

#### Assumption 1a

*Student midwives with children perceive significantly higher levels of stress than student midwives without children.*

#### Assumption 1b

*Student midwives with children apply maladaptive coping behaviors significantly more often and adaptive behaviors significantly less often than student midwives without children.*

### Work-privacy conflict among student midwives

The construct “work-privacy conflict” describes two possible causal directions in the interplay between work and private life: Thus, work-related stressors may spill over and have a negative impact on private life (or vice versa) [32]. Maintaining a work-life balance or balancing both study-related and personal demands were also identified as stressors in midwifery studies [19, 23, 24]. Among student midwives who had completed a clinical internship, work-privacy conflict was associated with lower intention to stay in the profession [33]. Students’ intention to stay in the study program was also influenced by observing that practicing midwives faced challenges in maintaining a work-life balance (caring for clients and own family) [34]. Furthermore, Australian studies on preferences of midwifery continuity of care as future employment model found that challenges related to family responsibilities and work-life balance were perceived among student midwives [35–37]. Although no studies to date exist on the link between work-privacy conflict and coping behaviors of student midwives, a literature overview on medical students’ well-being indicated that students with family demands may experience role conflict and guilt, and dealing with conflicting demands may be associated with maladaptive coping behaviors such as eating disorders or substance abuse [38]. Therefore, according to the current state of research, there is a research gap on the associations between work-privacy conflict, stress perception, and coping behaviors of student midwives in Germany. Furthermore, it is unclear whether parenthood and potential work-privacy conflicts moderate the relationship between stress perception and coping behaviors due to a higher proportion of student midwives with children (compared to other study programs) [25, 26]. We explore following assumptions:

#### Assumption 2

*Student midwives’ work-privacy conflict is positively associated with stress perception (2a) and maladaptive coping behaviors (2b), and negatively associated with adaptive coping behaviors (2c).*

#### Assumption 3

*Student midwives’ work-privacy conflict (3a) and parenthood (3b) moderate the association between stress perception and coping behaviors.*

### Perceived impact of COVID-19 pandemic on studies

Midwifery care could not be postponed during the COVID-19 pandemic, unlike routine medical procedures, and remained an indispensable component of healthcare. Maternity staff, like other health professionals (e.g., nurses), engaged in close physical contact with pregnant and childbearing women, making them a vulnerable group during the pandemic [39]. A scoping review by Schmitt and colleagues (2021) illustrated that the COVID-19 pandemic had a negative impact on maternity staff’s mental health. They faced various challenges (among others, efforts to cope with challenges while experiencing high fear of contagion, struggling with overwork and exhaustion, experiencing challenges in coping with ethical-moral dilemmas, learning to cope with women’s anxiety and loneliness), resulting in increased depression, anxiety, stress levels, and risk of post-traumatic stress symptoms [39]. Another study found that the strongest predictors of psychological distress among midwives and nurses during the pandemic were home and family stress [40].

Similarly, few studies with student midwives also indicated a negative impact of the COVID-19 pandemic on students’ mental health: an increase in stress levels, depression, anxiety, perceptions of isolation, intention to leave [41–45], and a decrease in psychosocial wellbeing and motivation to study [42]. Students were found to be concerned with adapting to new learning and teaching conditions, worried about course progression and their careers [42], as well as infecting their own families or patients, and faced particular challenges when infections occurred [46]. Furthermore, student midwives reported to feel expendable in terms of their value and contribution, as e.g. personal protective equipment was not always available to them. Students were dissatisfied with hospital and university communication, as it was experienced confusing and inconsistent. Witnessing perceived compromised midwifery care during the pandemic was experienced as emotional burden. Private aspects such as household obligations and financial worries remained problematic [45]. Based on current research, hardly any studies have been conducted on the relationship between student midwives’ stress perception and coping behaviors during the COVID-19 pandemic. A qualitative study from Turkey suggested that student midwives coped within the pandemic as follows: distracting leisure activities, sleep, increased social media use but also spending time with family [46]. In contrast, a scoping review on stressors and coping strategies among nursing students showed that the COVID-19 pandemic was experienced distressing, with students using coping strategies such as seeking information and consultation, staying optimistic and transference [47]. Furthermore, a repeated-measures study with nursing students found that both stress perception and coping strategy scores were higher during COVID-19 pandemic (compared to before the pandemic) and were significantly associated with each other [48]. Studies with health professionals (i.a. midwives) indicated on the one hand that negative coping increased during the COVID-19 pandemic [49] and on the other hand that coping strategies such as social support or avoidance were risk factors, whereas positive attitudes towards the stressful situation was the main protective factor [50]. Based on current research, it is currently unknown how German student midwives perceive the impact of the COVID-19 pandemic on their studies and how it is related to their stress perception and coping behaviors. We therefore explore following assumptions:

#### Assumption 4

*Student midwives’ perception of the impact of the COVID-19 pandemic on studies is positively associated with stress perception.*

#### Assumption 5

*Student midwives’ perception of the impact of the COVID-19 pandemic on studies moderates the association between stress perception and coping behaviors.*

#### Assumption 6

*Student midwives’ work-privacy conflict moderates the association between perception of the impact of the COVID-19 pandemic on studies and stress perception.*

**Fig. 1:**
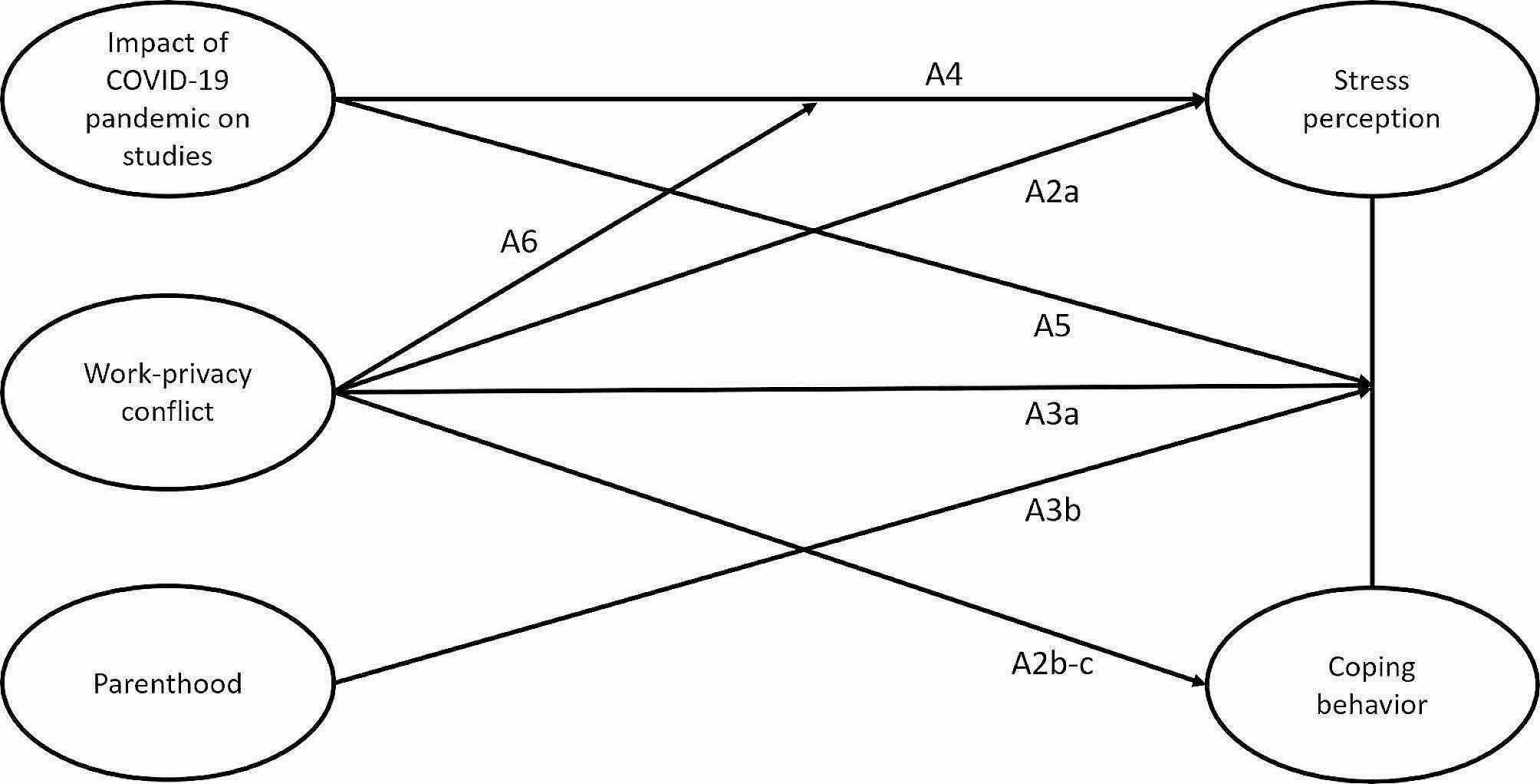
Conceptual model with assumptions

A conceptual model of all formulated assumptions is provided in Fig. 1.

### Study aims

Based on current state of research and the transactional stress model [10], this study aims to examine the links between stress perception, coping behaviors and work-privacy conflict of student midwives in Germany. Thereby, we aimed to assess the role of parenthood, work-privacy conflict and perceived impact of COVID-19 pandemic on studies in these associations. Hence, an overarching research question for the present study follows: How are student midwives’ stress perceptions, coping behaviors, work-privacy conflicts, and perceptions of COVID-19 pandemic impact on their studies in northern Germany associated? Hereby we aim to address aforementioned research gaps.

## Methods

### Study design and recruitment process

The “Healthy MidStudents” study was designed as a cross-sectional online survey for student midwives in northern Germany. Data collection took place between October 2022 and January 2023. Inclusion criteria for study participation was enrollment in the study program midwifery science in northern Germany (see Additional file 1 for further information) from the 2nd semester onwards, thus having practical experience in their studies. Recruitment was conducted by consent of program directors of nine universities in northern Germany. At the time of the survey, about 560 students were enrolled at nine universities in five federal states (Bremen, Hamburg, Mecklenburg-Western Pomerania, Lower Saxony and Schleswig-Holstein) in northern Germany. A pre-test was conducted with three midwives and research assistants prior to recruitment to review branching, skip logics, comprehension and duration. The researchers (CJ.A. and I.E.) scheduled recruitment appointments with the universities to present this study during lectures and to invite for voluntary, anonymous participation by students. At five universities, in-person recruitment was not possible for seven cohorts (out of 19 cohorts in total) due to organizational reasons. Here, the study was either presented in video conferences or the information on study participation was sent by e-mail to student midwives. A reminder for conducting the survey was sent to students via universities over the course of recruitment. Accordingly, the majority of students were reached during their theoretical study stage. For students who were reached only by e-mail, setting during participation could not be controlled. The “Healthy MidStudents” study was pre-registered at OSF [51] and covered data collection on student midwives’ occupational health literacy, health behaviors, stress perception, coping behaviors, work-privacy conflict, perception of impact of COVID-19 pandemic, and workplace health promotion. This article presents results on stress perception, coping behaviors, work-privacy conflict, and perception of impact of COVID-19 pandemic. Another article will report findings on health literacy and health behaviors. This survey was developed for this study and has not been previously published elsewhere (see Additional file 2).

## Variables

### Sociodemographic and study-related variables

For sociodemographic data collection, self-constructed items on age, parenthood, number of children, highest level of education, migration background, body weight and height were used, as well as validated scales. Gender was assessed using the item of the Copenhagen Psychosocial Questionnaire (COPSOQ III) [52]. Marital status was obtained using the item from a national survey, Mikrozensus 2023 [53]. Likewise, for study-related data, self-constructed items were included to survey participants’ study location, training level (number of semesters) and type of study program (primary, supplementary or post qualifying). Primary qualifying study program is entered by novice students following the reform of the German Midwifery Act, the supplementary qualifying study program by students who started vocational training before the reform and continued their midwifery education at a university. Post qualifying study program is designed for practicing midwives who have already completed their vocational training.

### Stress perception

To assess stress perception, the German version of the 10-item Perceived Stress Scale (PSS-10) was used [54, 55]. The items refer to perceived stress within the past month (e.g., “In the last month, how often have you been upset because of something that happened unexpectedly?”) and are rated on a 5-point Likert scale (0 = *never*, 1 = *almost never*, 2 = *sometimes*, 3 = *fairly often*, 4 = *very often*). Four positively stated items (items 4, 5, 7, and 8) were reversed prior to analysis. PSS-10 total scores were calculated by summing all 10 items. Given that the PSS-10 was not developed for diagnostic purposes and no cut-off values were defined, higher values describe higher levels of stress. The German version of the scale used was identified as a reliable (Cronbach’s α = 0.84), valid (CFI = 0.96; TLI = 0.95; RMSEA = 0.07), and economic instrument for assessing perceived stress [55]. This instrument was selected due to its internationally widespread use and establishment as a self-report scale for measuring stress perception, its evident associations with an increased health risk [55] as well as satisfactory psychometric properties for the German population [56] and university students [57]. In addition, two self-constructed items were applied to measure stress perception during two study stages (theoretical and practical) using a 5-point Likert scale (0 = *very low*, 1 = *low*, 2 = *moderate*, 3 = *high*, 4 = *very high*). The theoretical study stage comprises teaching in the classroom at universities in theory (lectures and seminars) and practice (skills lab), while the practical study stage comprises professional training in real-life work environment, namely clinical and outpatient settings [58].

### Coping behaviors

Coping behavior was measured using the 20-item scale of the Stress and Coping Inventory (SCI) [59]. Four items each describe a coping strategy, four scales out of five represent adaptive coping behaviors: 1) positive thinking (e.g., “I tell myself that stress and pressure also do have their good parts.“), 2) active stress coping (e.g., “I do everything I can to prevent stress in the first place.“), 3) social support (e.g., “When I feel pressure, I have people to help me.“), 4) support in faith (e.g., “When I feel stress and pressure, I find support in faith.“), and one scale represent maladaptive coping behaviors: 5) alcohol and cigarette consumption (e.g., “When things become too overwhelming for me, I sometimes grab the bottle.“). All items are rated on a 4-point Likert scale (1 = *does not apply*, 2 = *rather does not apply*, 3 = *rather applies*, 4 = *applies exactly*). One item of the scale alcohol and cigarette consumption (“No matter how much stress I get, I would never turn to alcohol or cigarettes because of stress.”) was reversed prior to analysis. Scale scores were calculated by summing up 4 items each. Higher values describe higher coping or, in the case of the maladaptive scale, higher maladaptive coping [60]. The SCI was validated (confirmation of factorial structure and significant correlations to stress symptoms) and considered reliable (Cronbach’s alpha between α = 0.74 and α = 0.88). Thereby, this instrument was selected due to its satisfactory psychometric properties and the documented links between coping strategies and stress-related symptoms [59] or stress [61].

### Work-privacy conflict

For the assessment of work-privacy conflict we used the 4-item scale of COPSOQ III, e.g., “The demands of my work interfere with my private and family life.” [52]. All items are rated on a 5-point Likert scale (1 = *to a very small extent*, 2 = *to a small extent*, 3 = *somewhat*, 4 = *to a large extent*, 5 = *to a very large extent*). Following the COPSOQ manual, ratings were transformed to point values of 0, 25, 50, 75 and 100, with mean values ranging from 0 (minimum) to 100 points (maximum). The instrument does not provide any cut-off values, thus higher values indicate a stronger work-privacy conflict [52]. COPSOQ represents an internationally widely used and well-established instrument for surveying psychosocial stress at work and was published as a new version in 2019 (COPSOQ III) [62]. In line with previous studies [32, 63], a new validation study confirmed overall good to very good psychometric properties, e.g. for the scale work-privacy conflict α = 0.92 [52].

### Perceived impact of COVID-19 pandemic on studies

Three self-constructed items were used to assess the students’ subjectively perceived impact of the COVID-19 pandemic on their studies. Students were first asked whether the COVID-19 pandemic had influenced their studies (three response options: 1 = *yes*, 2 = *no*, 3 = *n. a.*). The second item assessed which aspects of the study program were affected (multiple choice answer options: *practical assignments during studies, lectures / seminars during studies, skills lab / internships during studies, contact with fellow students, contact with lecturers / dean’s office* incl. free-text field for more precise information). The third item surveyed how the COVID-19 pandemic has affected their studies. It is rated on a 5-point Likert scale (0 = *very negatively*, 1 = *rather negatively*, 2 = *moderate*, 3 = *rather positively*, 4 = *very positively*).

### Data analysis

In total, over 560 student midwives (all enrolled students at the nine universities in northern Germany) were contacted. The online survey homepage was visited 540 times. The survey was completed by *n* = 343 participants (61.3% of contacted students). Data was checked for plausibility, outliers, mechanical response tendencies and missing values. Since there were 0.5% missing values (according to rule of thumb less than 5%) in our data set, the analysis was performed without imputations. Testing the hypothesis that the data are MCAR also yielded insignificant results by Little’s test (null hypothesis that data is MCAR could not be rejected) [64]. In order to maintain a complete dataset, pair- and listwise deletion was applied. One participant was excluded from data analyses due to implausible values, as this participant displayed an exclusion criterion (indication: 1st semester), resulting in a final sample size of *n* = 342. Outliers’ effect on the regression analysis was checked by calculating and reporting cook’s distance. Single items were recoded, where necessary, and scales were built. Descriptive analysis of the data was performed, including correlation analyses for main variables. Data was checked for normal distribution using skewness, kurtosis as well as histograms and Q-Q plots. Due to partly non-normally distributed data and large differences in sample sizes between groups, Mann-Whitney U-Tests as a non-parametric test were executed for testing assumption 1. A post-hoc sensitivity analysis using adjusted *p*-values according to Holm-Bonferroni were performed for Mann-Whitney-U-Test results for differences between student midwives with and without children. Moreover, Spearman’s correlation coefficients with bootstrapped confidence intervals were calculated. Further prerequisites for linear regression and moderation analyses were tested. Linearity, normal distribution of residuals, homoscedasticity and autocorrelation between adjacent residuals were checked graphically. To control for heteroscedasticity and non-normally distributed data, bootstrapping was performed. Multicollinearity was tested by using variance inflation factor (VIF). After all main associations were examined, moderation analyses were conducted separately by using centered interaction terms of concerned variables. Grand mean centering was performed for construction of products only for continuous variables. According to Cohen, *f*^2^ was classified as a small (0.02), medium (0.15) and large (0.40) effect size. Cohen’s *d* was classified as a small (0.20), medium (0.50) and large (0.80) effect size, and *R*^2^ as a small (0.02), medium (0.13) and large (0.26) explained variance [65]. All statistical analyses were performed with IBM® SPSS® Statistics (version 26, IBM, Armonk, NY, USA). Moderation analyses were conducted using Hayes’ PROCESS macro version 4.2 for SPSS. In addition, free-text responses were inductively analyzed and interpreted using Qualitative Content Analysis by Mayring [66] using MAXQDA Plus for Qualitative Data Analysis (version 20.0.6, 2020, VERBI GmbH, Berlin, Germany). The first author carried out the coding and analysis (IE), and the category structure and interpretation were discussed by members of the research team until consensus was reached.

## Results

### Characteristics of study sample

Table 1 presents sociodemographic and study-related variables of the sample. In summary, of 342 participants (response rate: 61.3%), the majority were female (*n* = 339), between 21 and 25 years old (*n* = 185), single (*n* = 288), had no migrant background (*n* = 302), and had high school graduation as their highest level of education (*n* = 195). A total of 51 participants had one or more children. When surveyed, most participants were either in their 3rd semester (*n* = 152) or 5th semester (*n* = 120) and were enrolled at a university in a primary qualifying degree program (*n* = 253). Whereas 81 participants were studying in a supplementary qualifying degree program at a college/university parallel to their midwifery training at a vocational school and seven participants were in a post-qualifying study program following their midwifery training at a vocational school.

**Table 1:** Sociodemographic and study-related variables of the sample

Variables	*n*	%
Gender	342	
Female	339	99.1
Male	1	0.3
Other	2	0.6
Age*	338	
≤ 20 years	35	10.3
21–25 years	185	54.0
26–30 years	71	20.8
31–35 years	27	7.9
36–40 years	10	3.0
≥ 41 years	10	3.0
Migration background	342	
Yes	40	11.7
No	302	88.3
Marital status	341	
Single	288	84.2
Married	45	13.2
Divorced	7	2.0
Registered partnership	1	0.3
Parenthood	342	
Children	51	14.9
No children	291	85.1
Highest level of education	342	
High school	195	57.0
Vocational training	84	24.6
Bachelor	43	12.6
Master	17	4.7
Diploma	3	0.9
PhD	0	0.0
Training level	341	
2nd semester	1	0.3
3rd semester	152	44.4
4th semester	27	7.9
5th semester	120	35.1
6th semester	10	2.9
7th semester	29	8.5
8th semester	1	0.3
Vacation semester	1	0.3
Type of study program	341	
Primary qualifying	253	74.0
Supplementary qualifying	81	23.7
Post-qualifying	7	2.0

*Note*. *M = 25.3, *SD* = 5.6, Range = 19-56

## Main results

### Stress perception, coping behaviors and work-privacy conflict of student midwives

Table 2 displays the characteristics of main variables, including means, standard deviations, ranges, minimum and maximum values and Cronbach’s alpha. Acceptable reliability was confirmed for the scales stress perception, coping behavior, and work-privacy conflict measured by Cronbach’s alpha (α > 0.70). Scores are comparable to those obtained in validation studies [52, 55, 59].

**Table 2:** Characteristics of main variables

Variables	M	SD	Range	Min	Max	α
Stress perception during theoretical study stage (*n* = 342)	2.42	0.84	0–4	0	4	/
Stress perception during practical study stage (*n* = 342)	3.12	0.83	0–4	0	4	/
Stress perception (*n* = 335) (Stress perception total scores)	2.22 (22.22)	0.66 (6.59)	0–4 (4–16)	0.6 (6)	4 (40)	0.89
Positive thinking^1^ (*n* = 338)	9.61	2.21	4–16	4	16	0.71
Active stress coping^1^ (*n* = 337)	10.72	2.01	4–16	4	16	0.73
Social support^1^ (*n* = 337)	13.76	2.19	4–16	4	16	0.83
Support in faith^1^ (*n* = 337)	7.47	2.69	4–16	4	16	0.76
Alcohol and cigarette consumption^2^ (*n* = 339)	5.79	2.33	4–16	4	16	0.75
Work-privacy conflict (*n* = 340)	66.10	19.88	0-100	6.25	100	0.87
Perceived impact of COVID-19 pandemic on studies (*n* = 314)	1.38	0.89	0–4	0	4	/

*Note*. *N* = 342, *M* = Mean, *SD* = Standard deviation, *Min* = Minimum, *Max* = Maximum, α = Cronbach’s alpha, ^1^adaptive coping behaviors, ^2^maladaptive coping behaviors

As shown in Table 2, student midwives’ overall stress perception within the past month was moderate on average (*n* = 335, *M* = 2.22, *SD* = 0.66), with higher levels of perceived stress reported by 13.4% of students (*n* = 45). Stress perception was rated higher on average during the practical study stage (*n* = 342, *M* = 3.12, *SD* = 0.83) than the theoretical study stage (*n* = 342, *M* = 2.42, *SD* = 0.84). Furthermore, mean scores indicated both social support (*n* = 337, *M* = 13.76, *SD* = 2.19) and active stress coping (*n* = 337, *M* = 10.72, *SD* = 2.01) as most prevalent coping behaviors in the present sample, whereas alcohol and cigarette consumption (*n* = 339, *M* = 5.79, *SD* = 2.33) was identified as least prevalent coping behavior. About 37.4% of students (*n* = 127) reported experiencing work-privacy conflicts to a (very) large extent, with the mean corresponding to a moderate to large extent (*n* = 340, *M* = 66.10, *SD* = 19.88). Table 3 illustrates Spearman’s correlation coefficients among main variables.

**Table 3:** Spearman’s correlation coefficients among main variables

Variables	1	2	3	4	5	6	7
1. Stress perception	–						
2. Positive thinking	− 0.41*** [-0.51, − 0.31]	–					
3. Active stress coping	− 0.13* [-0.24, − 0.03]	0.07 [-0.06, 0.19]	–				
4. Social support	− 0.37*** [-0.47, − 0.27]	0.17** [0.05, 0.28]	0.02 [-0.09, 0.13]	–			
5. Support in faith	− 0.14** [− 0.25, − 0.03]	0.20*** [0.09, 0.30]	0.05 [-0.07, 0.17]	0.09 [-0.04, 0.21]	–		
6. Alcohol and cigarette consumption	0.09 [-0.03, 0.20]	0.04 [-0.07, 0.15]	− 0.01 [-0.12, 0.10]	− 0.14** [-0.25, − 0.02]	− 0.07 [-0.18, 0.04]	–	
7. Work-privacy conflict	0.51*** [0.42, 0.60]	− 0.24*** [-0.36, − 0.13]	0.02 [-0.11, 0.14]	− 0.26*** [-0.36, − 0.16]	− 0.15** [-0.26, − 0.04]	0.08 [-0.04, 0.21]	–
8. Perceived impact of COVID-19 pandemic on studies	0.00 [-0.10, 0.10]	0.12* [-0.00, 0.24]	− 0.01 [-0.13, 0.11]	− 0.04 [-0.15, 0.06]	0.04 [-0.09, 0.18]	− 0.05 [-0.17, 0.05]	− 0.09 [-0.19, 0.03]

*Note*. *n* = 299. One-tailed Spearman correlation coefficients were used. *p <.05. **p <.01, ***p <.001. BCa Bootstrap 95% CIs reported in brackets, confidence intervals based on 1000 bootstrap samples

### Differences between student midwives with and without children

Table 4 illustrates the results of Mann-Whitney-U-Tests for differences between student midwives with and without children. Assumption 1a assumed that student midwives with children perceive higher levels of stress than student midwives without children. No statistically significant difference was found for stress perception. Thus, assumption 1a must be rejected. Assumption 1b assumed that student midwives with children apply maladaptive coping behaviors more often and adaptive coping behaviors less often than student midwives without children. No statistically significant difference was found regarding the use of maladaptive coping behaviors (alcohol and cigarette consumption) between students with and without children. Statistically significant differences were evident regarding the use of adaptive coping behaviors between students with and without children. Therefore, student midwives without children reported significantly higher levels of social support (Mean rank = 173.51, *Mdn* = 14.00) than students with children (Mean rank = 143.12, *Mdn* = 13.00), *U* (N1 = 287, N2 = 50) = 5881.00; *z* = -2.08; *p* =.04; *d* = 0.22. According to Cohen [65], this value corresponds to a small effect size. No statistically significant differences were found for positive thinking, active stress coping and support in faith. Thus, assumption 1b can only be partially accepted. A post-hoc sensitivity analysis using adjusted *p*-values according to Holm-Bonferroni revealed no statistically significant results (see Table 4).

**Table 4:** Mann-whitney-U-tests of stress perception and coping behaviors for students with and without children

Variables	Students with children		Students without children		U	z	p	Adjusted p
	n	Mean rank	n	Mean rank				
Stress perception (*n* = 335)	50	166.66	285	168.24	7058.00	-0.11	0.92	> 0.999
Positive thinking^1^ (*n* = 338)	50	193.89	288	165.27	5980.50	-1.93	0.05	0.25
Active stress coping^1^ (*n* = 337)	50	188.27	287	165.64	6211.50	-1.54	0.13	0.52
Social support^1^ (*n* = 337)	50	143.12	287	173.51	5881.00	-2.08	0.04*	0.24
Support in faith^1^ (*n* = 337)	51	167.55	286	169.26	7219.00	-0.12	0.91	> 0.999
Alcohol and cigarette consumption^2^ (*n* = 338)	51	164.57	287	170.38	7067.00	-0.42	0.68	> 0.999

*Note*. *N* = 342, *U* = Mann-Whitney-U, *z* = z-statistics, *p* = asymptotic significance, adjusted *p* = adjusted *p*-values according to Holm-Bonferroni, *p <.05, **p <.01, ***p <.001, ^1^adaptive coping behaviors, ^2^maladaptive coping behaviors

### Relationships between stress perception, coping behaviors and work-privacy conflict

Assumption 2 assumed that student midwives’ work-privacy conflict is positively associated with stress perception (2a) and maladaptive coping behaviors (2b), and negatively associated with adaptive coping behaviors (2c). Linear regression analyses were conducted to test assumption 2. Results revealed that work-privacy conflict significantly predicted stress perception (*ß* = 0.53, *p* =.001) and explained about 28% of its variance, indicating a large effect size (*f*^2^ = 0.62) [65] (Table 5). The associated regression model was significant (*F*_(1,331)_ = 128.01, *p* <.001). Thus, assumption 2a can be accepted. Second, work-privacy conflict significantly predicted alcohol and cigarette consumption as maladaptive coping behaviors (*ß* = 0.14, *p* =.015, *R*^2^ = 0.02). Again, the model was significant (*F*_(1,334)_ = 6.78, *p* <.01) with *f*^2^ = 0.14, indicating a medium effect size [65] (Additional file 3). Thus, assumption 2b can be accepted. Third, work-privacy conflict significantly predicted following adaptive coping behaviors: positive thinking (*ß* = − 0.25, *p* =.001, *R*^2^ = 0.06) and social support (*ß* = − 0.23, *p* =.001, *R*^2^ = 0.05). Regression models were significant (positive thinking: *F*_(1,334)_ = 22.96, *p* <.001; social support: *F*_(1,333)_ = 18.19, *p* <.001) (Additional file 3). Calculated effect sizes were between medium to large (positive thinking: *f*^2^ = 0.25; social support: *f*^2^ = 0.23) [65]. Linear regression analysis of the association between work-privacy conflict and active stress coping (*ß* = − 0.01, *p* =.932, *R*^2^ = 0.00) as well as between work-privacy conflict and support in faith (*ß* = − 0.11, *p* =.075, *R*^2^ = 0.01) were not significant, leading to a partial acceptance of assumption 2c (Additional file 3).

**Table 5:** Linear regression analysis of association between work-privacy conflict and stress perception

Variable	b	SE	ß	p
Constant	1.05 (0.86, 1.26)	0.10		*p* =.001
Work-privacy conflict	0.02 (0.01, 0.02)	0.00	0.53	*p* =.001
*n*	333			
*R* ^2^	0.28***			
Adj. *R*^2^	0.28***			

*Note*. All values have been rounded off to two decimal places except p-values. *p <.05; **p <.01. ***p <.001. 95% bias corrected and accelerated confidence intervals reported in parentheses. Confidence intervals, p-values and standard errors based on 1000 bootstrap samples. Cook’s distance was used to examine outliers (between 0.000 and 0.052). *b* = unstandardized coefficient; *SE* = standard error; *β* = standardized coefficient

To test assumption 3 (student midwives’ work-privacy conflict (3a) and parenthood (3b) moderate the associations between stress perception and coping behaviors), moderation analyses based on model 2 were conducted. Overall models were significant for all coping behavior scales (positive thinking: *F*_(5,324)_ = 17.23, *p* <.001, *R*^2^ = 0.23; active stress coping: *F*_(5, 324)_ = 3.73, *p* =.003, *R*^2^ = 0.07; social support: *F*_(5, 324)_ = 13.16, *p* <.001, *R*^2^ = 0.15; support in faith: *F*_(5, 324)_ = 2.40, *p* =.037, *R*^2^ = 0.03; alcohol and cigarette consumption: *F*_(5, 324)_ = 2.39, *p* =.037, *R*^2^ = 0.04; Additional file 4). No significant main effects nor moderation effects were found except for maladaptive coping behaviors. Work-privacy conflict moderated the effect between stress perception and alcohol and cigarette consumption significantly, Δ*R*² = 1.34%, *F*_(1,324)_ = 4.53, *p* =.034, 95% CI [0.00, 0.04] (Additional file 4). Thus, assumption 3a can be partly accepted and assumption 3b must be rejected.

### Perceived impact of COVID-19 pandemic on studies

The vast majority of student midwives perceived an overall impact of the COVID-19 pandemic on their studies (*n* = 315, 92.1%), 55.6% (*n* = 190) reported a negative impact compared to 9.4% (*n* = 32) and 26.9% (*n* = 92) who reported a positive and moderate impact, respectively. Among multiple choice answers, most participants stated that lectures and seminars (*n* = 296, 86.5%) as well as contact with fellow students (*n* = 249, 72.8%) were affected. Approximately half of the participants further identified COVID-19 specific influences on skills lab and/or internships (*n* = 199, 58.2%), practical assignments during studies (*n* = 170, 49.7%), and contact with lecturers and/or dean’s office (*n* = 162, 47.4%).

In free-text fields, about *n* = 86 student midwives (25.1%) provided further information on aspects of study program that were perceived to be impacted by the COVID-19 pandemic. In summary, qualitative analysis indicated that the shift to online teaching (lectures and seminars) had both positive (e.g., improved work-life balance) and negative effects (e.g., organizational and technical problems, reduced contact with fellow students) on studies. Furthermore, perceived negative impacts of the COVID-19 pandemic were reported regarding the practical aspects of the study program. Students reported a shortage of skills lab and limited possibilities for practical assignments, with profound consequences for vocational training (e.g. knowledge acquisition deficits, insecurity, frustration and increased stress). In particular, the challenges (such as hygiene regulations, or role conflict) of caring for childbearing women during the COVID-19 pandemic were mentioned. Further information on the qualitative analysis of free-text responses is presented in Table 6.

**Table 6:** Free-text responses on the impact of COVID-19 pandemic on studies

Aspect of study program	Details on impact of COVID-19 pandemic
Shift to online/hybrid teaching (*n* = 62)	Positive aspects: • work-life balance (e.g. childcare) • no travel time and cost savings • easier attendance at lectures • stress reduction • flexibility • easier health-promoting exercise in home office • possibility to work at one’s own learning pace Negative aspects: • problems with coordination and implementation of curriculum adaptation without deficits • uncertainties and overload among teaching staff • limitations due to technical problems • reduction and cancellation of lectures • challenges with self-study • lower effectiveness of online teaching compared to classroom teaching • worries about missing important exams due to potential COVID-19 infection
Shortage of skills lab (*n* = 19)	• knowledge acquisition deficits • insecurity • disappointment • frustration • partial implementation of follow-up dates unrelated to present teaching content
Reduced contact with fellow students (*n* = 28)	• difficulties in establishing contact, cooperation, group formation, and cohesion • loneliness at study beginning, especially for students who had moved for study • profound impacts over the course of the study program
Changes at practical assignments (*n* = 20)	• limitations of practical assignments • exclusion in practical assignments due to students’ own illness leading to increased stress • difficulties in achieving target number of cases for admission to the midwifery team • fewer instructions by teaching staff • fewer opportunities for exchange within the team • unclear regulations due to changing political guidelines as well as different approaches by teams • increased workload and stress due to pandemic-related additional job tasks and short-staffing • difficulties in care of childbearing women due to COVID-19 pandemic: no permission for students to attend childbearing women with COVID-19 infection, role conflict due to taking on an additional support person role for the birthing woman in the course of stricter hygiene regulations at hospitals (attendants not allowed), distressing perception of more distant care of COVID-19-infected patients in the maternity hospital
COVID-19 hygiene regulations (*n* = 15)	• impact of obligation to cover mouth and nose on health (e.g. skin problems, headaches in the course of work) and practice (e.g. more difficult communication with families, limited perception of non-verbal communication) • higher stress levels due to perceived pressure by universities and employers regarding vaccination status • disadvantages for unvaccinated or not fully vaccinated students in practical training • wish to have had more time for vaccination (for using later developed vaccines)

*Note*. *n* = 86

### Relationships between perceived impact of COVID-19 pandemic on studies and stress perception

Assumption 4 assumed a positive association between student midwives’ perception of the impact of the COVID-19 pandemic on studies and their stress perception. Linear regression analysis of this association was not significant (*F*_(1,306)_ = 0.44, *p* >.05), leading to rejection of assumption 4 (Table 7).

**Table 7:** Linear regression analysis of association between perception of impact of COVID-19 pandemic and stress perception

Variable	b	SE	ß	p
Constant	2.18 (2.05, 2.32)	0.07		*p* =.001
Perceived impact of COVID-19 pandemic on studies	0.03 (-0.05, 0.11)	0.04	0.04	*p* =.514
*n*	308			
*R* ^2^	0.00			
Adj. *R*^2^	− 0.00			

Note. All values have been rounded off to two decimal places except p-values. *p <.05; **p <.01. ***p <.001. 95% bias corrected and accelerated confidence intervals reported in parentheses. Confidence intervals, p-values and standard errors based on 1000 bootstrap samples. Cook’s distance was used to examine outliers (between 0.000 and 0.060). *b* = unstandardized coefficient; *SE* = standard error; *β* = standardized coefficient

To test assumptions 5 and 6, moderation analyses based on model 1 were conducted. It was assumed that student midwives’ perception of the impact of the COVID-19 pandemic on studies moderates the association between stress perception and coping behaviors (assumption 5). Overall models of moderation analyses were significant for only two adaptive coping behaviors (positive thinking: *F*_(3, 301)_ = 25.99, *p* <.001, *R*^2^ = 0.22; social support: *F*_(3,301)_ = 16.18, *p* <.001, *R*^2^ = 0.18). The examination of main effects revealed that stress perception was negatively related to positive thinking (*b* = -1.55, *t*(305) = -8.56, *p* <.001, 95% CI [-1.88, -1.20]) and social support (*b* = -1.40, *t*(305) = -6.81, *p* <.001, 95% CI [-1.79, -0.98]) as well as perception of impact of COVID-19 pandemic on studies was positively related to positive thinking (*b* = 0.27, *t*(305) = 1.98, *p* =.048, 95% CI [0.02, 0.53]). However, no significant interaction effects were identified (Additional file 5). Thus, assumption 5 must be rejected. Assumption 6 suggested that student midwives’ work-privacy conflict moderates the association between perception of the impact of the COVID-19 pandemic on studies and stress perception. The overall model of assumption 6 was significant, (*F*_(3,302)_ = 42.18, *p* <.001), predicting 28.39% of the variance. Yet, moderation analysis results revealed a positive association between work-privacy conflict and stress perception (*b* = 0.02, *t*(306) = 11.01, *p* <.001, 95% CI [0.01, 0.02]), but no significant moderation effect of work-privacy conflict on the relationship between perception of the impact of the COVID-19 pandemic and stress perception, Δ*R*² = 0.49%, *F*_(1,302)_ = 2.16, *p* =.142, 95% CI [-0.00, 0.01] (Table 8). Thus, assumption 6 also must be rejected.

**Table 8:** Moderation analysis for association between perception of impact of COVID-19 pandemic, work-privacy conflict and stress perception

Variable	b	SE	t	p
Constant	2.22 (2.16, 2.28)	0.03	70.08	*p <*.001
Perceived impact of COVID-19 pandemic on studies (centred)	0.03 (-0.04, 0.10)	0.04	0.83	*p* =.405
Work-privacy conflict (centred)	0.02 (0.01, 0.02)	0.00	11.01	*p <*.001
Perceived impact of COVID-19 pandemic on studies x work-privacy conflict	0.00 (-0.00, 0.01)	0.00	1.47	*p* =.142
*n*	306			
*R* ^2^	0.28***			

Note. All values have been rounded off to two decimal places except p-values. *p <.05; **p <.01. ***p <.001. 95% bootstrap confidence intervals reported in parentheses. Confidence intervals and standard errors based on 5000 bootstrap samples. Cook’s distance was used to examine outliers (Perceived impact of COVID-19 pandemic: between 0.000 and 0.060; work-privacy conflict: between 0.000 and 0.052). *b* = unstandardized coefficient; *SE* = standard error

## Discussion

To the best of our knowledge of the current state of research, this is the first study that examined the associations between stress perception, coping behaviors, work-privacy conflict and perceptions of COVID-19 pandemic impact on studies of student midwives in Germany. Overall, our exploratory analysis was able to offer initial study results that provide a basis for future research.

### Stress perception, coping behaviors and work-privacy conflict

Descriptive results showed that student midwives in northern Germany, on average, experienced moderate levels of stress within the past month. Since most of data collection was carried out during theoretical study stage, results on stress perception were interpreted in this context. The comparison of stress perception between theoretical and practical stages provided indications on higher stress levels during practical stages. Furthermore, most prevalent coping behaviors among student midwives in the present study were social support and active stress coping, while alcohol and cigarette consumption was identified as least prevalent coping behavior.

The current state of research on student midwives’ stress perception reveals that few international pre-pandemic studies exist, with differences in methodology and inconsistencies in results [8]. Thus, on the one hand, a study of 150 bachelor student midwives from the UK yielded a similar mean and standard deviation using also PSS-10 instrument [27]. On the other hand, other studies from India [20], Iran [15], UK [12] and Ireland [31] using different instruments reported high stress levels in the samples between 40 and 60%. Similarly, two recent studies conducted during the COVID-19 pandemic in Australia and Iran, and thus more comparable to the setting of our sample, showed also varying prevalence of stress in their cross-sectional surveys [41, 43]. Both studies used the Depression, Anxiety and Stress Scales (DASS-21) instrument [67] and reported that while 40.2% of Australian nursing and midwifery students demonstrated moderate to severe symptoms of stress and 25% severe to extremely severe symptoms [41], in contrast, the majority of Iranian students demonstrated normal mental health status with only 15% of midwifery students reporting mild symptoms of stress [43]. Thus, there is not yet clear evidence on the prevalence of high stress levels among student midwives. Additionally, according to current state of research, there remains a research gap regarding student midwives’ mental health and well-being at different time periods during their studies as well as different settings [8]. Based on current knowledge, it remains questionable why moderate stress levels were identified among student midwives: both individual factors (e.g. resilience or socially desirable response behavior oriented towards job demands) and organizational factors (e.g. favorable study conditions during theoretical stage as well as potential differences in stress perceptions between theoretical and practical study stages) may be influencing variables. In literature to date, the working conditions during theoretical and practical study stages have not been compared with regard to student midwives’ stress perception. However, many stressors are known in the clinical setting that students encounter during the practical study stage, including high emotional job demands [e.g., 15–17], challenges in work organization such as on-call, time pressure, high workload [e.g., 18, 20, 21] as well as challenging social relationships in the workplace and unsupportive work culture [e.g., 16, 22]. Few studies also shed light on stressors in the academic setting of student midwives, e.g. aspects of learning environments in higher education institutions [19].

Furthermore, comparing present results on coping behaviors to the current state of research illustrates that previous studies also identified active or problem-focused coping [13, 30] and seeking emotional or social support [13, 30, 31] as most commonly used coping behaviors among nursing and midwifery students. In contrast to present descriptive findings, another study showed that risk behaviors such as alcohol consumption were prevalent in student midwives, among others [29]. Moreover, students displaying risk behaviors demonstrated higher levels of stress and predominantly engaged in passive coping behaviors such as escape avoidance [29]. According to current state of research, there are still too few studies with student midwives on their coping behaviors, as studies to date refer mostly to samples with nursing students [9].

For this study, it was assumed that differences in stress perception and coping behaviors exist between student midwives with and without children. The proportion of students with children in the present sample (14.9%) was similar to that in a previous survey of German midwifery trainees and students in 2017 (15.7%) [25]. Results from our sample did not show differences in stress perception and coping behaviors, except for social support. As presumed, student midwives without children reported significantly higher levels of social support than students with children. Contrary to expectations, student midwives with children scored higher on positive thinking than students without children (result was at significance threshold). However, post-hoc sensitivity analyses revealed no statistically significant group differences. Despite higher demands among students with children, our results indicated that student midwives with and without children do not differ greatly in terms of their stress perception and coping behaviors. At present, there are no other studies that have compared student midwives with and without children regarding their stress perception and coping behaviors. Yet, contrary to our results, previous studies indicated that family responsibilities were a stressor for student midwives [14, 18] and was considered a challenge in terms of midwifery continuity of care as a future employment model [35–37]. Also a qualitative study with Australian nurses and midwives identified difficulties in coordinating child care and work in terms of work scheduling, access to leave and casual work, as well as with negative impacts on private relationships and finances [68]. In a literature overview, Dunn and colleagues argued that medical students with family responsibilities may increase the risk for poor health outcomes and maladaptive coping behaviors such as eating disorders or substance abuse [38].

Consistent with assumptions, our study results showed that work-privacy conflict positively predicted student midwives’ stress perception and alcohol and cigarette consumption, and negatively predicted positive thinking and social support. Overall, interpretation of these results should be conservative, as the regression analyses yielded very low explanations of variance (except for stress perception). No relationship was found between work-privacy conflict and active stress coping, support in faith. Contrary to assumptions, parenthood and work-privacy conflict did not moderate the association between stress perception and coping behaviors. Although a significant interaction effect was identified for work-privacy conflict and stress perception on alcohol and cigarette consumption, a reasonable interpretation should be discussed due to a very small Δ*R*² and a confidence interval close to zero. Nevertheless, our findings suggest that work-privacy conflict is a substantial stressor for student midwives and is associated with their stress perception and coping behavior.

Again, no other studies to date examined the associations between stress perception, coping behaviors, work-privacy conflict, and parenthood of student midwives. However, prior studies indicated that maintaining a work-life balance or balancing study-related and personal demands were experienced as stressors by student midwives [19, 23, 24]. In addition, studies showed that a lower intention to remain in the study or profession was associated with students’ own work-privacy conflict post clinical placements [33] as well as observing the challenges of practicing midwives to maintain a work-life balance (caring for clients and own family) [34]. Furthermore, studies with midwives highlighted a risk for high levels of work-privacy conflict for this profession [21, 68, 69] and thus a potential negative spill-over of stress into non-work life [68]. Inflexible shift schedules, staff shortages, and very high workloads were challenges for taking recreational leave or hindered work-life balance [68]. High levels of work-privacy conflict were also attributed to midwives’ high sense of commitment at work [70] or to the fact that this profession is predominantly a female one, with women often carrying out a higher proportion of the care and domestic work in families [68]. A potential reason for increased work-privacy conflict among student midwives in our sample may be due to the special features of the dual study program in midwifery science in Germany (see Additional file 1), especially in context of study-related changes caused by COVID-19 pandemic. Recent academization in Germany increased the study requirements of the profession and led to major changes in the education system. These academic challenges coupled with straining working conditions in midwifery profession may result in a concentration of stressors and thus increased work-privacy conflict for student midwives.

### Perceived impact of COVID-19 pandemic on studies

According to descriptive study results, most student midwives perceived an overall impact of COVID-19 pandemic on their studies and about half of our study sample considered it as negative. The majority identified impacts of the COVID-19 pandemic on lectures and seminars and contact with fellow students, and about half of the participants on skills lab and/or internships, practical assignments during studies, and contact with lecturers and/or dean’s office. Further, qualitative content analysis of free-text responses provided additional information on COVID-19 pandemic induced changes and challenges on studies, revealing five main themes: ‘shift to online/hybrid teaching’, ‘shortage of skills lab’, ‘reduced contact with fellow students’, ‘changes at practical assignments’ and ‘COVID-19 hygiene regulations’. In contrast to expectations, statistical analyses of the present study did not indicate an association between student midwives’ perception of the impact of the COVID-19 pandemic on studies and their stress perception. Although descriptive and qualitative results of this study may suggest increased stress levels among student midwives during the COVID-19 pandemic, no direct contrast with statistical analyses is warranted. Qualitative results should rather be seen as an informative supplement to statistical analyses outlining students’ specific challenges during COVID-19 pandemic. Additionally, the information in the free-text fields was only answered by part of the sample (25.1% of students) and thus does not represent the entire sample. Moreover, statistical results may also stem from a biased assessment of stress perception, e.g. social desirability (see limitations in Chap. 4.5.). In comparison to current state of research, previous studies indicated a negative impact of the COVID-19 pandemic on the mental health of maternity staff [39] and student midwives [41–46], including increased depression, anxiety, and stress levels. However, previous quantitative studies did not examine perceptions of the COVID-19 pandemic in relation to student midwives’ mental health. Thus, the scoping review by Schmitt et al. (2021) referred to studies that examined midwives’ stress perception during the COVID-19 pandemic compared to before the pandemic or compared to other professions or the general population [39]. Yet, the review presented a study from Australia that showed that more COVID-19-related concerns were associated with higher levels of stress [71]. The quantitative cross-sectional studies with student midwives also did not operationalize perceptions of the COVID-19 pandemic nor examined this in relation to students’ mental health outcomes. Instead these studies assessed the prevalence of depression, anxiety and stress levels of student midwives during the pandemic and/or compared them between two measurement points during the pandemic, between two samples, to the general population or analyzed them in relation to socio-demographic characteristics [41, 43, 44]. In contrast, a longitudinal study on the impact of the changes and challenges caused by the COVID-19 pandemic on stress perception of different student groups at one university in northern Germany showed no significant differences between 2019 and 2020 (before and during the pandemic) [57]. In comparison to the present study, about 85% of the students surveyed indicated that the pandemic had impacted their studies and showed significantly higher stress levels than students who perceived no impact on their studies [57]. Furthermore, qualitative analyses of three studies with student midwives yielded similar results on the influence of the COVID-19 pandemic on studies and mental health, e.g. higher stress perception or reduced motivation [42, 45, 46]. Compared to qualitative analysis results of present study, previous studies also showed positive impacts resulting from the shift to online teaching, such as flexibility in teaching [42], easier access to resources related to courses, no transportation problems and being with own families [46]. In contrast, further positive aspects mentioned in the study of Rasmussen and colleagues (2022) included improved study techniques, quality of teaching strategies and building of resilience [42]. Similar to our study, previous studies also indicated that face-to-face classes were reduced [45] or less lesson hours were offered [46], technical difficulties existed [46], hospital and university communication was experienced as confusing and inconsistent [45], self-study as challenging (too much time in front of the computer, concentration problems, too much homework) [46], and worries about course progression and own career existed [42]. It was also found that reduced social interaction with fellow students and faculty was experienced as challenging and lonely [42, 45, 46]. Similarly, challenges in care of childbearing women due to COVID-19 pandemic were reported in a previous study by Kuliukas et al. (2021). Student midwives from Australia reported feeling expendable in terms of their value and contribution as they were sometimes excluded from clinical situations, and felt letting down women when they were unable to be present at women’s births. Witnessing perceived compromised midwifery care was experienced as emotionally stressful. They experienced an impaired relationship with women due to restrictions and wearing personal protective equipment [45]. Unlike the present study, students from previous studies reported concerns about infecting their own families or patients leading to personal distress and anxiety [45, 46]. Overall, the discussion of the current state of research in comparison to present analysis results illustrates that there is still an inconsistent research evidence regarding the perceived impact of the COVID-19 pandemic on the studies and mental health of student midwives. There are also many methodological discrepancies between existing studies and our study, hampering the comparability of results.

Furthermore, statistical analyses of the present study showed that perception of the impact of the COVID-19 pandemic on studies did not moderate the association between stress perception and coping behaviors. Nor did work-privacy conflict moderate the association between perception of the impact of the COVID-19 pandemic on studies and stress perception. However, by identifying significant main effects between work-privacy conflict and stress perception and between stress perception and positive thinking and social support (adaptive coping strategies), these relationships may indicate independence from the perceived impact of the COVID-19 pandemic.

Compared to current state of research, only one further study was conducted on the relationship between student midwives’ stress perception and coping behaviors during the COVID-19 pandemic. This qualitative study described how student midwives from Turkey coped with the disadvantages of distance teaching in the COVID-19 pandemic by using coping behaviors, such as distracting leisure activities or increased media use [46]. Although some quantitative studies of nursing students or health professionals have shown that stress and negative coping increased during the pandemic (compared to before the pandemic) [48, 49], there are currently no studies that have assessed perceptions of the influence of the COVID-19 pandemic and in relation to student midwives’ stress perception, coping behaviors and work-privacy conflict. Nonetheless, prior studies suggested that student midwives with work-privacy conflicts are more psychologically distressed during the COVID-19 pandemic. Two qualitative studies indicated that competing responsibilities affected students’ ability to focus and engage in learning: financial worries (such as losing employment), worries about children (e.g. problems associated with ‘home schooling’), elderly parents and isolation from geographically distant family and friends [42, 45]. Another study from the Netherlands and Belgium revealed that having children was found to be positively related to students’ depression level [44]. Lastly, a qualitative study from United Kingdom demonstrated higher stress among midwives due to compounded workforce pressures during COVID-19 pandemic such as staffing shortages but also difficulties in maintaining a work-life balance [72].

### Implications for future research

The present study represents a pilot study in northern Germany that does not allow generalization to the German population of student midwives. There is therefore a need for a larger, Germany-wide survey. Given the current limited and heterogeneous body of research on student midwives’ mental health (e.g., the prevalence of high stress levels) and their coping behaviors [8, 9], it is recommended for future research to use validated scales and to consider different time points during studies (e.g. at the beginning of studies, during studies, before graduation, at job start) and settings (e.g. during theoretical and practical study stages, including clinical and outpatient settings). In this regard, comparative studies (e.g., comparing cohorts, study groups, or in comparison to practicing midwives) and longitudinal studies (e.g., examining student midwives over the course of their studies until entering practice) are of particular interest. With regard to surveying future settings that are particularly challenging, such as that of a pandemic, future research should use validated instruments assessing perceptions of these. Further, research is recommended on the causal and influencing factors as well as consequences of distress and work-privacy conflict in student midwives. Due to the distinctive study program (i.a. equal percentage split of teaching at the university and in professional practice) and cohort composition (i.a. higher proportion of students with children than in other study programs as well as students on the second educational path), research on the prevalence and perceived impact of work-privacy conflicts, including students with children, is also recommended. Taken together, these recommendations may enable a better understanding of the particular study and work requirements of midwifery students, their causes and consequences. Last, there is a need for interventional studies that promote student midwives’ mental health, as a review by Oates and colleagues (2019) has identified only three interventional studies with student midwives to date [8]. Here, study curricula that prepare students for professional life are conceivable, including competence training as an integrative element. Overall, future research should be interdisciplinary and incorporate insights from various disciplines such as psychology, sociology, midwifery science or educational research, e.g. in order to examine how different training approaches and concepts relate to students’ mental health and practical skills. In addition to interdisciplinary research teams, the involvement of diverse stakeholders (e.g., educators, student midwives’ families) is also recommended in order to gain more holistic perspectives, e.g. impacts of students’ work-privacy conflicts on their studies and private life. Cross-cultural or cross-contextual comparative studies could also provide helpful insights for international studies comparing education in midwifery.

### Implications for policy and practice

In order to reduce high stress levels among student midwives on a structural and individual level, a variety of approaches are needed. Implications for policy, practice, and university teaching are presented as follows.

First, legal frameworks created by governments, policy makers and regulatory authorities provide the basis for structurally shaping working conditions of midwives and consequently student midwives. These should focus, amongst others, on workforce planning, implementation of continuity of care or caseloading, increase in clinical autonomy, adequate remuneration and insurance regulations of self-employed midwives [73]. Improving midwives’ working conditions is of greatest relevance also in light of the demonstrated negative relationship between work-related well-being or health of healthcare workers and patient safety outcomes [74]; for example, midwife-led continuity models clearly had beneficial effects on women and babies [75].

Second, responsible institutions for practical training but also managing personnel of clinical and outpatient facilities should bear responsibility in designing health-promoting working conditions of student midwives, since half of study program comprises professional training in real-life work environment. Thus, the aim should be to reduce job demands and strengthen job resources in order to structurally improve working conditions of midwives as well as student midwives during clinical placements. For example, the introduction of flexible rostering systems may have positive effects on students’ work-life balance and mental health [76]. Furthermore, it is recommended to offer students comprehensive and ongoing support for transition to practice, both in the training of practical skills but also in personal development for interprofessional collaboration and building self-confidence [77, 78]. Also, a review on student retention in programs of nursing and midwifery education was able to show that sufficient support for students seems to be crucial [79]. Especially in uncertain times like the COVID-19 pandemic, it is recommended to offer support from academic staff and clinical mentors, implement uniform regulations and communicate risk management clearly and timely in order to reduce students’ stress levels [42, 71].

Third, universities should continuously improve study conditions and develop curricula. Overall, universities should consider special needs of their study cohorts (e.g. students with dependent children) and obtain regular feedback on study programs and organization as well as communicate changes transparently. For example, family-friendly services should be offered during studies (e.g. free childcare), but also more flexibility in terms of study content and exams. Furthermore, it is recommended that positively rated changes in studies caused by the COVID-19 pandemic should also be maintained for the post-pandemic period. In this way, hybrid teaching may be maintained in order to offer students increased flexibility during their studies that allows them to balance their study, family, and employment responsibilities [42]. Last, regarding behavioral prevention, it is recommended to implement workplace health promotion measures in order to develop students’ self-care competencies or resilience during studies [29]. Because midwifery care can involve stressful and sometimes traumatic work experiences, training courses on stress prevention and adaptive coping should be offered. Initial behavioral prevention interventions for stress management indicated that mindfulness training or educational programs may improve adaptive coping skills [9, 13, 80, 81] as well as student support circles or self-care workshops may improve resilience and self-care [8].

### Strengths and limitations

One of the strengths of our study is a response rate of 61.3%, which allowed us to assume that the results are representative for the population of student midwives in northern Germany. Our recruitment strategy and support of the cooperating universities enabled an increase in students’ motivation to participate. Thus, researchers were allowed to present the study predominantly in-person during lectures, and in some cases teaching time was given off for voluntary study participation. Teaching staff and study coordinators shared study information with student midwives. Personal contact and the possibility to ask questions directly to researchers during recruitment made it possible to reduce participation barriers. Additionally, in the interest of transparency and science communication, students were informed that they will receive study results and may participate in an online presentation of results at the end of the study project. The use of an online survey also allowed all students to participate flexibly in terms of location and time. Numerous detailed responses from participants via free-text fields indicated a great interest in the research topic. In addition, our sample varied in socio-demographic characteristics, e.g., age, parenthood, training level and type of study program. Another strength of the study is the use of validated instruments with satisfactory psychometric properties– also in comparison with other studies [32, 52, 55, 56, 59, 61, 63]. A further strength of the study is the interdisciplinary collaboration in the research project between psychologists, midwives, nutritionists and occupational physicians, allowing diverse professional perspectives on study design and interpretation of results. Overall, the study results offer initial insights on this so-far-unexplored topic in Germany.

However, there are also some limitations in this study. Besides using validated scales, self-developed items were used for perceptions of COVID-19 impact on studies since, to the researcher’s knowledge, there were no existing validated instruments for the research interest. Consequently, self-developed items might not adequately capture the research interest. Furthermore, interpretations of the results need to consider that most students were surveyed during theoretical study stages (due to the recruitment strategy via universities). Present descriptive study results on stress perception during practical and theoretical study stages suggest that study results may differ depending on study stage. No pre-post comparison was performed, so data may be retrospectively biased. In addition, it was not feasible to ensure that all study cohorts received the same recruitment approach. For most cohorts, a personal presentation of the study including time for study conduct was allowed. Due to organizational reasons, for some cohorts no time for study conduct was provided and for others only a digital approach was possible. These unintended recruiting inconsistencies were evident in the response rates of cohorts. Results may also be biased by, e.g., the presence of researchers and teaching staff during study conduct, although interest in true subjective assessment was emphasized by researchers. Accordingly, social desirability and possible errors of judgment cannot be ruled out by the self-assessment of participants. Student midwives may be exposed to a professional image based on experiences during studies and practice that suggests that stress is part of the profession. These underlying assumptions may also have led to a biased assessment of stress perception. Last, there is a lack of research, which is why present study results were also discussed with regard to related study programs, and should be interpreted appropriately.

## Conclusions

To the best of our knowledge of the current state of research, this study presents initial results on German student midwives’ stress perception, coping behaviors, work-privacy conflict and perceptions of COVID-19 pandemic impact on studies. The COVID-19 pandemic also displayed specific contextual conditions that have also not been previously surveyed in student midwives in Germany. Key findings highlighted that student midwives, on average, reported moderate stress levels during theoretical study stage, with most engaging in adaptive coping behaviors. Results suggested that stress perception was higher during practical stage, thus indicating differing demands for student midwives between study stages as well as a need for further research in the future on the prevalence of high stress levels among student midwives. Furthermore, students with higher levels of work-privacy conflict were positively associated with stress perception and maladaptive coping behaviors, and negatively associated with adaptive coping behaviors. Due to the distinctive study program (i.a. equal percentage split of teaching at the university and in professional practice) and cohort composition (i.a. higher proportion of students with children than in other study programs), research on the prevalence and perceived impact of work-privacy conflicts on stress perception as well as greater consideration in practice is recommended. Contrary to expectations, statistical moderation analyses did not indicate associations between student midwives’ stress perception, coping behaviors, work-privacy conflict, and perception of COVID-19 pandemic impact on studies. Yet, qualitative results provided additional information on COVID-19 pandemic induced changes and challenges on studies. Future research should address the development of validated instruments to capture particularly challenging settings (such as a pandemic) while practice should ensure sufficient support for students. This study provides a basis for future research, in which, among other things, a longitudinal examination of student midwives’ stress perception and coping behaviors should be conducted at different time points and settings, as well as investigating causal-effect relationships. Based on results, implications for policy, practice and university teaching are derived. Key recommendations address the improvement of working conditions in practice settings as well as implementation of workplace health promotion measures in order to develop student midwives’ self-care competencies or resilience.

### Electronic supplementary material

Below is the link to the electronic supplementary material.

Supplementary Material 1

Supplementary Material 2

Supplementary Material 3

Supplementary Material 4

Supplementary Material 5

## Data Availability

No data are available. The datasets analyzed during the current study are not publicly available due to German national data protection regulations. The datasets used and analyzed during the current study are available from the corresponding author on reasonable request.
