# Supplementary material for: Stress perception, coping behaviors and work-privacy conflict of student midwives in times of COVID-19 pandemic: the “Healthy MidStudents” study in Germany
Source: BMC Health Serv Res. 2024 May 7;24:594. doi: 10.1186/s12913-024-10823-5 (PMC11075225; doi:10.1186/s12913-024-10823-5)
Supplement: Supplementary file 1 — Supplementary Material 1 [file 12913_2024_10823_MOESM1_ESM.pdf]

Additional file 1. Further information on study program midwifery science in Germany.

Since the reform of the German Act on the study and profession of midwives (HebG) in 2020, the theoretical training of midwives in Germany is no longer provided at vocational schools, but at universities and corresponds to a bachelor's degree. In a dual education system, the training of practical competencies takes place in clinical and outpatient settings. A distinctive feature of the study program - compared to other medical or healthcare-related study programs - is the equal percentage split of teaching at the university and in professional practice, with at least 2200 hours each (§ 11, German Act on the study and profession of midwives (HebG)). Academization is intended to address the increasing complexity of obstetric care. In an effort to address staffing shortages and rising birth rates (1), the number of student midwives has been increased (2, 3). In Germany, approximately 3,175 midwifery science study seats were offered at 223 hospitals in 2022 (4). The reform has led to the establishment of new training sites and cooperative agreements with hospitals at many locations.

References:

1. Statistisches Bundesamt. Veränderung der Zahl der Lebendgeborenen zum jeweiligen Vorjahr. 2022. https://www.destatis.de/DE/Themen/Gesellschaft-Umwelt/Bevoelkerung/Geburten/Tabellen/lebendgeborene-differenz.html;jsessionid=1B734444DB290728D791E8F90FE95042.live721. Accessed 19 June 2023.
2. Statistisches Bundesamt. Gesundheit. Grunddaten der Krankenhäuser 2018. 2020. https://www.destatis.de/DE/Themen/Gesellschaft-Umwelt/Gesundheit/Krankenhaeuser/Publikationen/Downloads-Krankenhaeuser/grunddaten-krankenhaeuser-2120611187004.pdf?__blob=publicationFile. Accessed 19 June 2023.
3. Statistisches Bundesamt. Gesundheit. Grunddaten der Krankenhäuser 2019. 2021. https://www.destatis.de/DE/Themen/Gesellschaft-Umwelt/Gesundheit/Krankenhaeuser/Publikationen/Downloads-Krankenhaeuser/grunddaten-krankenhaeuser-2120611197004.pdf;jsessionid=E02AD119258AC5DD721FCD96AEE08ECA.live741?__blob=publicationFile. Accessed 19 June 2023.
4. Statistisches Bundesamt. Grunddaten der Krankenhäuser 2022. 2023. https://www.destatis.de/DE/Themen/Gesellschaft-Umwelt/Gesundheit/Krankenhaeuser/Publikationen/Downloads-Krankenhaeuser/statistischer-bericht-grunddaten-krankenhaeuser-2120611227005.html. Accessed 24 January 2024.
