# Supplementary material for: Stress perception, coping behaviors and work-privacy conflict of student midwives in times of COVID-19 pandemic: the “Healthy MidStudents” study in Germany
Source: BMC Health Serv Res. 2024 May 7;24:594. doi: 10.1186/s12913-024-10823-5 (PMC11075225; doi:10.1186/s12913-024-10823-5)
Supplement: Supplementary file 2 — Supplementary Material 2 [file 12913_2024_10823_MOESM2_ESM.pdf]

Additional file 2. List of main variables and measurement.

Construct (type of variable)	Measurement	Number of items
Sociodemographic data	Self-developed items on age, parental status, number of children, highest level of education, migration background. Item on gender from Copenhagen Psychosocial Questionnaire (COPSOQ III) (Lincke et al., 2021) and item on marital status from Mikrozensus 2023 questionnaire (Statistische Ämter des Bundes und der Länder, 2023)	7
Study-related data	Self-developed items on study location, training level, study program	3
General health		
Health status	Copenhagen Psychosocial Questionnaire (COPSOQ III) (Lincke et al., 2021)	1
Body-Mass-Index	Body weight and height (BMI)	2
Health behaviors		
Dietary pattern	Mediterranean Diet Score (MDS), Western Diet Score (WDS) (Stewart et al., 2016)	10
Physical activity	International Physical Activity Questionnaire – short form (IPAQ) (Craig et al., 2003)	7
Sleep quality	Pittsburgh Sleep Quality Index (PSQI-D) (Hinz et al., 2017)	1
Smoking	Self-developed items on smoking status	2
	Fagerström Test for Nicotine Dependence (FTND) (Heatherton et al., 1991)	6
Alcohol consumption	Alcohol Use Disorders Identification Test - Consumption (AUDIT-C) (Ganz et al., 2017)	3
Comparison between practical and theoretical study stage	Self-developed items	3
Occupational Health literacy	Occupational Health Literacy Scale (OHLS) (Friedrich et al., 2023)	12
Stress perception	Perceived Stress Scale (PSS-10) (Klein et al., 2016)	10
	Self-developed item on comparison between practical and theoretical study stage	1
Work-privacy conflict	Copenhagen Psychosocial Questionnaire (COPSOQ III) (Lincke et al., 2021)	4
Coping behaviors	Stress- and Coping Inventory (SCI) (Satow, 2012)	20

Preparation for profession	Self-developed items on health counselling competences, preparation for midwifery profession and interprofessional collaboration, preferred job activities	4
Perceived impact of COVID-19 pandemic on studies	Self-developed items on perceived impact of COVID-19 pandemic on studies	3
Workplace health promotion	Self-developed items on knowledge, use and evaluation of workplace health promotion offers in academic institutions and in the workplace, reasons for non-participation, needs for further workplace health promotion offers	25
