# Supplementary material for: Stress perception, coping behaviors and work-privacy conflict of student midwives in times of COVID-19 pandemic: the “Healthy MidStudents” study in Germany
Source: BMC Health Serv Res. 2024 May 7;24:594. doi: 10.1186/s12913-024-10823-5 (PMC11075225; doi:10.1186/s12913-024-10823-5)
Supplement: Supplementary file 3 — Supplementary Material 3 [file 12913_2024_10823_MOESM3_ESM.pdf]

Additional file 3. Linear regression analysis of associations between work-privacy conflict and coping behaviors.

	Positive thinking				Active stress coping				Social support			
**Variable**	***b***	**SE**	***ß***	***p***	***b***	**SE**	***ß***	***p***	***b***	**SE**	***ß***	***p***
Constant	11.51 (10.62, 12.44)	0.45		*p* = .001	10.77 (9.92, 11.53)	0.41		*p* = .001	15.42 (14.54, 16.32)	0.45		*p* = .001
Work-privacy conflict	-0.03 (-0.04, -0.02)	0.01	-.25	*p* = .001	-0.00 (-0.01, 0.01)	0.01	-.01	*p* = .932	-0.03 (-0.04, -0.01)	0.01	-.23	*p* = .001
n	336				335				335			
R^2	.06***				.00				.05***			
Adj. R^2	.06***				-.00				.05***			

Additional file 3. *Continued.*

	Support in faith				Alcohol and cigarette consumption			
**Variable**	***b***	**SE**	***ß***	***p***	**b**	**SE**	***ß***	***p***
Constant	8.48 (7.32, 9.75)	0.58		p = .001	4.68 (3.83, 5.45)	0.42		p = .001
Work-privacy conflict	-0.02 (-0.03, -0.00)	0.01	-.11	p = .075	0.02 (0.00, 0.03)	0.01	.14	p = .015
n	335				336			
R^2	.01*				.02**			
Adj. R^2	.01*				.02**			

Note. All values have been rounded off to two decimal places except p-values. *p < 0.05; **p < 0.01. ***p < 0.001. 95% bias corrected and accelerated confidence intervals reported in parentheses. Confidence intervals, p-values and standard errors based on 1000 bootstrap samples. Cook's distance was used to examine outliers (Positive thinking: between 0.000 and 0.049; active stress coping: between 0.000 and 0.049; social support: between 0.000 and 0.192; support in faith: between 0.000 and 0.090; alcohol and cigarette consumption: between 0.000 and 0.079). b = unstandardized coefficient; SE = standard error; β = standardized coefficient.
