# Supplementary material for: Stress perception, coping behaviors and work-privacy conflict of student midwives in times of COVID-19 pandemic: the “Healthy MidStudents” study in Germany
Source: BMC Health Serv Res. 2024 May 7;24:594. doi: 10.1186/s12913-024-10823-5 (PMC11075225; doi:10.1186/s12913-024-10823-5)
Supplement: Supplementary file 4 — Supplementary Material 4 [file 12913_2024_10823_MOESM4_ESM.pdf]

Additional file 4. Moderation analyses for associations between stress perception, work-privacy conflict, parental status and coping behaviors.

	Positive thinking				Active stress coping				Social support			
**Variable**	***b***	**SE**	***t***	***p***	***b***	**SE**	***t***	***p***	***b***	**SE**	***t***	***p***
Constant	10.24 (9.14, 11.26)	0.54	18.45	$p < .001$	11.42 (10.39, 12.42)	0.52	21.38	$p < .001$	12.84 (11.74, 13.96)	0.56	22.41	$p < .001$
Stress perception (centred)	-1.14 (-2.52, 0.33)	0.73	-1.50	$p = .135$	-0.90 (-2.61, 0.95)	0.89	-0.97	$p = .334$	-1.38 (-2.80, 0.18)	0.75	-1.77	$p = .077$
Work-privacy conflict (centred)	-0.00 (-0.02, 0.01)	0.01	-0.62	$p = .539$	0.01 (-0.00, 0.03)	0.01	1.70	$p = .089$	-0.01 (-0.03, 0.00)	0.01	-1.36	$p = .176$
Stress perception x Work-privacy conflict	0.01 (-0.01, 0.03)	0.01	1.41	$p = .161$	0.02 (-0.00, 0.03)	0.01	1.69	$p = .091$	-0.00 (-0.02, 0.02)	0.01	-0.35	$p = .727$
Parenthood	-0.38 (-0.91, 0.19)	0.29	-1.27	$p = .207$	-0.44 (-0.97, 0.13)	0.28	-1.53	$p = .128$	0.53 (-0.06, 1.12)	0.30	1.75	$p = .082$
Stress perception x Parenthood	-0.19 (-0.99, 0.58)	0.41	-0.46	$p = .648$	0.05 (-0.93, 0.96)	0.47	0.10	$p = .919$	0.17 (-0.69, 0.96)	0.41	0.40	$p = .690$
n	330				330				330			
R^2	.23***				.07**				.15***			

Additional file 4. *Continued.*

	Support in faith				Alcohol and cigarette consumption			
**Variable**	***b***	**SE**	***t***	***p***	**b**	**SE**	***t***	***p***
Constant	7.78 (6.25, 9.31)	0.77	9.85	$p < .001$	5.66 (4.10, 7.40)	0.84	6.68	$p < .001$
Stress perception (centred)	1.47 (-0.88, 3.70)	1.15	1.24	$p = .215$	0.04 (-2.16, 2.48)	1.18	0.03	$p = .974$
Work-privacy conflict (centred)	-0.01 (-0.03, 0.01)	0.001	-1.00	$p = .317$	0.01 (-0.00, 0.03)	0.01	1.74	$p = .083$
Stress perception x Work-privacy conflict	0.00 (-0.02, 0.03)	0.01	0.15	$p = .882$	0.02 (0.00, 0.04)	0.01	2.13	$p = .034$
Parenthood	-0.17 (-0.98, 0.65)	0.41	-0.40	$p = .690$	0.01 (-0.89, 0.82)	0.44	0.01	$p = .989$
Stress per-ception x Parenthood	-1.03 (-2.17, 0.18)	0.60	-1.66	$p = .098$	0.17 (-1.10, 1.31)	0.62	0.27	$p = .787$
n	330				330			
R^2	.03*				.04*			

Note. All values have been rounded off to two decimal places except *p*-values. *$p < 0.05$; **$p < 0.01$. ***$p < 0.001$. 95% bootstrap confidence intervals reported in parentheses. Confidence intervals and standard errors based on 5000 bootstrap samples. Cook's distance was used to examine outliers (Positive thinking: between 0.000 and 0.098; active stress coping: between 0.000 and 0.061; social support: between 0.000 and 0.118; support in faith: between 0.000 and 0.048; alcohol and cigarette consumption: between 0.000 and 0.255). *b* = unstandardized coefficient; SE = standard error.
