# Supplementary material for: Stress perception, coping behaviors and work-privacy conflict of student midwives in times of COVID-19 pandemic: the “Healthy MidStudents” study in Germany
Source: BMC Health Serv Res. 2024 May 7;24:594. doi: 10.1186/s12913-024-10823-5 (PMC11075225; doi:10.1186/s12913-024-10823-5)
Supplement: Supplementary file 5 — Supplementary Material 5 [file 12913_2024_10823_MOESM5_ESM.pdf]

Additional file 5. Moderation analyses for associations between stress perception, perception of impact of COVID-19 pandemic on studies and coping behaviors.

	Positive thinking				Active stress coping				Social support			
**Variable**	***b***	**SE**	***t***	***p***	***b***	**SE**	***t***	***p***	***b***	**SE**	***t***	***p***
Constant	9.61 (9.38, 9.82)	0.11	86.73	$p < .001$	10.71 (10.48, 10.92)	0.11	94.41	$p < .001$	13.80 (13.58, 14.03)	0.11	121.16	$p < .001$
Stress perception (centred)	-1.55 (-1.88, -1.20)	0.17	-8.56	$p < .001$	-0.54 (-0.95, -0.14)	0.21	-2.56	$p = .011$	-1.40 (-1.79, -0.98)	0.21	-6.81	$p < .001$
Perceived impact of COVID-19 pandemic on studies (centred)	0.27 (0.02, 0.53)	0.13	1.98	$p = .048$	0.01 (-0.23, 0.24)	0.12	0.10	$p = .952$	-0.18 (-0.42, 0.10)	0.13	-1.36	$p = .176$
Stress perception x perceived impact of COVID-19 pandemic on studies	0.09 (-0.38, 0.53)	0.24	0.34	$p = .735$	0.02 (-0.38, 0.41)	0.20	0.10	$p = .920$	0.18 (-0.19, 0.60)	0.20	0.89	$p = .375$
n	305				305				305			
R^2	.22***				.03				.18***			

Additional file 5. *Continued.*

	Support in faith				Alcohol and cigarette consumption			
**Variable**	***b***	**SE**	***t***	***p***	**b**	**SE**	***t***	***p***
Constant	7.42 (7.14, 7.71)	0.15	48.99	$p < .001$	5.80 (5.54, 6.07)	0.13	42.80	$p < .001$
Stress perception (centred)	-0.47 (-0.92, -0.06)	0.22	-2.18	$p = .030$	0.47 (0.06, 0.91)	0.21	2.16	$p = .031$
Perceived impact of COVID-19 pandemic on studies (centred)	0.20 (-0.16, 0.53)	0.18	1.12	$p = .262$	-0.14 (-0.49, 0.21)	0.17	-0.75	$p = .455$
Stress perception x perceived impact of COVID-19 pandemic on studies	0.12 (-0.40, 0.59)	0.25	0.45	$p = .654$	-0.01 (-0.57, 0.61)	0.30	-0.05	$p = .963$
n	305				305			
R^2	.02				.02			

Note. All values have been rounded off to two decimal places except *p*-values. *$p < 0.05$; **$p < 0.01$. ***$p < 0.001$. 95% bootstrap confidence intervals reported in parentheses. Confidence intervals and standard errors based on 5000 bootstrap samples. Cook's distance was used to examine outliers (Positive thinking: between 0.000 and 0.098; active stress coping: between 0.000 and 0.061; social support: between 0.000 and 0.118; support in faith: between 0.000 and 0.048; alcohol and cigarette consumption: between 0.000 and 0.255). *b* = unstandardized coefficient; SE = standard error.
